# Protective Effects of Human Nonrenal and Renal Stromal Cells and Their Conditioned Media in a Rat Model of Chronic Kidney Disease

**DOI:** 10.1177/0963689720965467

**Published:** 2020-12-01

**Authors:** Barbara Imberti, Domenico Cerullo, Daniela Corna, Cinzia Rota, Monica Locatelli, Anna Pezzotta, Martino Introna, Chiara Capelli, Claudia Elisa Carminati, Ton J. Rabelink, Danielle G. Leuning, Carlamaria Zoja, Marina Morigi, Giuseppe Remuzzi, Ariela Benigni, Valerie Luyckx

**Affiliations:** 1 Department of Molecular Medicine, Istituto di Ricerche Farmacologiche Mario Negri IRCCS, Bergamo, Italy; 2 Laboratory of Cell Therapy “G. Lanzani”, Azienda Socio Sanitaria Territoriale (ASST) Papa Giovanni XXIII, Bergamo, Italy; 3 Department of Internal Medicine, Leiden University Medical Centre, Leiden, Holland; 4 “L. Sacco” Department of Biomedical and Clinical Science, University of Milan, Milan, Italy; 5 Institute of Biomedical Ethics and History of Medicine, University of Zurich, Zurich, Switzerland; 6 Renal Division, Brigham and Women’s Hospital, Harvard Medical School, Boston, MA, USA; * Both the authors are co-first author

**Keywords:** stromal cells, renal perivascular cells, conditioned medium, renal repair, chronic kidney disease

## Abstract

Mesenchymal stromal cells (MSCs) are emerging as a novel therapeutic option for limiting chronic kidney disease progression. Conditioned medium (CM) containing bioactive compounds could convey similar benefits, avoiding the potential risks of cell therapy. This study compared the efficacy of nonrenal and renal cell-based therapy with the corresponding CM in rats with renal mass reduction (RMR). Infusions of human kidney stromal cells (kPSCs) and CM-kPSCs, but not umbilical cord (uc) MSCs or CM-ucMSCs, reduced proteinuria and preserved podocyte number and nephrin expression in RMR rats. Glomerular fibrosis, microvascular rarefaction, and apoptosis were reduced by all treatments, while the peritubular microvascular loss was reduced by kPSCs and CM-kPSCs treatment only. Importantly, kPSCs and CM-kPSCs reduced NG2-positive pericytes, and all therapies reduced α-smooth muscle actin expression, indicating reduced myofibroblast expansion. Treatment with kPSCs also significantly inhibited the accumulation of ED1-positive macrophages in the renal interstitium of RMR rats. These findings demonstrate that the CM of ucMSCs and kPSCs confers similar renoprotection as the cells. kPSCs and CM-kPSCs may be superior in attenuating chronic renal injury as a cell source.

## Introduction

Chronic kidney disease (CKD) is predicted to become the fifth leading global cause of years of life lost by 2040^1–4^, and so developing novel therapies to prevent the progression of CKD is essential. Stromal cell-based therapy, which may repair damaged renal tissue, is a promising strategy for treating CKD^5–7^. Mesenchymal stromal cells (MSCs) are immature multipotent cells that can self-renew, form clonal populations, and differentiate into mesodermal tissue. MSCs can be obtained from bone marrow (bm), adipose tissue, the umbilical cord (uc), and connective tissue. Initial studies have demonstrated the renoprotective effects of different populations of MSCs, including bmMSCs and ucMSCs in experimental acute kidney injury (AKI) induced by glycerol, cisplatin, gentamicin, and ischemia-reperfusion injury^8–14^. Recently, a new population of human kidney perivascular stromal cells (kPSCs) obtained from surgically discarded human kidneys was characterized^15^. These cells have a transcriptional profile that is partially similar to that of human bm-MSCs and retain tissue-specific signatures of kidney development^15^. In initial studies, treatment with human kPSCs was found to significantly improve renal function in mice with glycerol-induced AKI^15^. While these are promising initial results with cell therapies in AKI, findings in AKI cannot be extrapolated directly to CKD.

The role of stem cells in slowing the progression of CKD has been investigated, but the effects have been inconsistent and variable^16,17^. A recent systematic review and meta-analysis of 71 animal studies concluded, however, that cell-based therapy could slow the development and progression of CKD, depending on cell type and route of administration^16^. We have previously described the therapeutic potential of human stromal cell populations of nonrenal and renal origin in a CKD model induced by adriamycin. Human bmMSCs, kPSCs, and, above all, ucMSCs reduced glomerulosclerotic and fibrotic lesions in this model^17^. Since MSCs and kPSCs did not incorporate into the kidney structure, attention has now shifted to investigating how MSCs enhance renal repair without differentiating into resident cells, so the paracrine activity of MSCs on endogenous renal cells has also been investigated^11,12,14,17^. The administration of conditioned medium (CM), which contains bioactive molecules and extracellular vesicles secreted by MSCs, could be a valid alternative to using parental cells to treat renal diseases^16,18,19^. Compared with therapy with parental cells, treatment with CM may have important medical advantages, including a superior safety profile, a lower risk of embolism, immunogenicity, genetic instability, and survival limitation^20^.

The aim of this study was to demonstrate the possible regenerative effects of human nonrenal and renal stromal cells and their corresponding CM in an established experimental model of CKD induced by renal mass reduction (RMR). The primary objective was to evaluate whether renal-derived kPSCs conferred greater nephroprotection than uc-MSCs in CKD; the second objective was to evaluate the effectiveness of the respective CM compared with cell therapy. We hypothesized that both cell types and their CM would exert protective effects against kidney injury and that CM would be confirmed as a potentially valid therapeutic option.

## Materials and Methods

### Human uc-MSCs

Human uc-MSCs (provided by Dr Martino Introna, Laboratory of Cell Therapy “G. Lanzani”, Azienda Ospedaliera Papa Giovanni XXIII, Bergamo, Italy) were derived from human ucs collected at the Obstetrics and Gynecology Unit, Papa Giovanni XXIII Hospital, Bergamo, Italy after cesarean section, as previously described^21^. The women undergoing cesarean section provided written informed consent for the use of their ucs for research purposes. All ucs were fully anonymized prior to being accessed. This procedure was approved by the ethics committee of the hospital (authorization n. 1239/2017).

The human uc-MSC phenotype was confirmed using flow cytometry. Human uc-MSCs expressed standard MSC markers such as CD73, CD90, and CD105 and were negative for the typical hematopoietic and endothelial cell markers CD31, CD34, and CD45. Human uc-MSCs were able to differentiate into osteocytes and chondrocytes and had a very low adipogenic potential^21^.

### Human kPSCs

Human kPSCs (provided by Dr A J Rabelink, Leiden University Medical Centre, Leiden, Holland) were isolated from human transplant-grade kidneys discarded for surgical reasons as previously described^15^. The study was approved by the local medical ethics committee at the Leiden University Medical Centre and the ethics advisory board of the STELLAR European Union consortium. Briefly, within 30 h of surgery, the renal artery was cannulated, and the kidney was perfused with collagenase and DNAse. After approximately 30 min, the tissue was digested, and the cell suspension was cultured and expanded in α-Minimum Essential Medium (αMEM) 1× Glutamax (Life Technologies Europe, Milan, Italy) containing 5% platelet lysate, glutamine, and penicillin/streptomycin. At passage 1, the kPSC population was isolated using magnetic-activated cell sorting based on NG2 expression^15^. The human kPSC phenotype was confirmed by flow cytometric analysis. In addition to NG2, kPSCs were positive for platelet-derived growth factor receptor-β, CD146, CD73, CD90, and CD105 and were negative for CD31, CD34, CD45, and CD56. Osteogenic and chondrogenic, but not adipogenic, differentiations were observed in kPSCs^15^.

CM was obtained by incubating human uc-MSCs or kPSCs for 15 h in αMEM 1× Glutamax under serum-free conditions. The CM was then centrifuged to remove cellular debris. The supernatant was transferred to Amicon Ultra-15 centrifugal Filter Devices with a cut-off of 3000 (Merck Millipore, Darmstadt, Germany) and centrifuged at 4000×*g* for 20 min to concentrate the volume of CM.

All uc-MSCs or kPSCs and their respective CM were harvested between the fourth and sixth passages and administered at a dose of 1.5 × 10^6^ cells/injection or as aliquots of CM (500 µl), derived from 1.5 × 10^6^ uc-MSCs or kPSCs.

### Rat Model of RMR

All procedures involving animals were performed in accordance with institutional guidelines in compliance with national (D.L.n.26, March 4, 2014) and international laws and policies (directive 2010/63/EU on the protection of animals used for scientific purposes) and were approved by the Institutional Animal Care and Use Committees of the Istituto di Ricerche Farmacologiche Mario Negri IRCCS and by the Italian Ministry of Health (approval number 1154/2015-PR).

Male Sprague-Dawley (SD) rats with initial body weights of 200 to 250 g were used for the experiments. Animals were housed at a constant temperature with a 12-h dark/12-h light cycle in a specific pathogen-free facility and fed a standard diet. RMR was achieved in anesthetized rats through the right nephrectomy and ligation of two or three branches of the left main renal artery (5/6 nephrectomy)^22^. Sham-operated rats underwent surgery but not RMR and served as controls. Postoperative analgesia was administered with buprenorphine (0.15 mg/kg). This RMR model is characterized by progressive proteinuria, glomerulosclerosis (GS), and tubulointerstitial fibrosis as a result of compensatory glomerular hemodynamic changes in response to nephron loss^22^.

**Figure 1.**
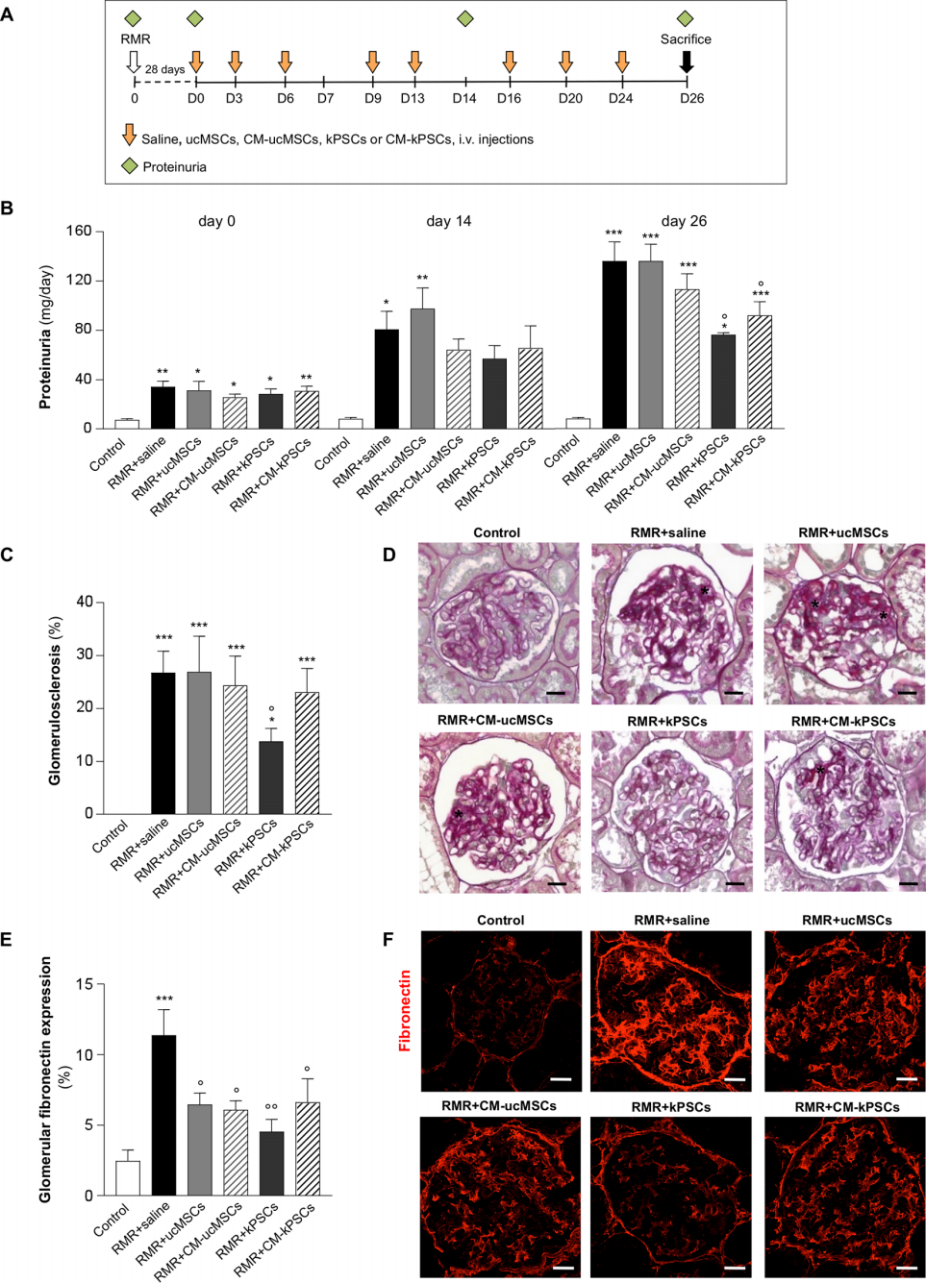
Effect of human ucMSCs, kPSCs, or their corresponding CM on proteinuria and glomerular structure in RMR rats. (A) Experimental design of *in vivo* studies. Rats with RMR after 28 days (day 0) received intravenous injections (i.v.) of saline, ucMSCs, kPSCs, or CM (1.5 × 10^6^ cells/rat, CM obtained from 1.5 × 10^6^ cells) at different time intervals. Animals were sacrificed at day 26. (B) Time course of proteinuria (day 0, 14, and 26) evaluated in control and RMR + saline, ucMSCs, kPSCs, or CM (*n* = 4 to 8 animals/group). Data are mean ± SE. *P < 0.05, **P < 0.01, and ***P < 0.001 versus control and °*P* < 0.05 versus RMR + saline at the corresponding time. (C) Quantification of the percentage of glomeruli affected by sclerotic lesions assessed in PAS-stained kidney sections of control and RMR rats receiving saline, ucMSCs, kPSCs, or CM on day 26. Data are mean ± SE. *P < 0.05, ***P < 0.001 versus control and °*P* < 0.05 versus RMR + saline (*n* = 4 to 8 animals/group). (D) Representative images of PAS-stained sections of kidneys from control and RMR rats receiving saline, ucMSCs, kPSCs, or corresponding CM. Asterisks indicate the area of glomerulosclerosis. Scale bars 20 μm. (E) Quantification of glomerular fibronectin staining evaluated in kidney sections of control and RMR rats receiving saline, ucMSCs, kPSCs, or CM on day 26. Data are mean ± SE. ***P < 0.001 versus control and °*P* < 0.05, °°*P* < 0.01 versus RMR + saline (*n* = 4 to 5 animals/group). (F) Representative images of glomerular fibronectin (red) in renal sections of control and RMR rats receiving saline, ucMSCs, kPSCs, or CM. Scale bars 25 μm. CM: conditioned medium; kPSCs: kidney perivascular stromal cells; PAS: periodic acid-Schiff; RMR: renal mass reduction; ucMSCs: umbilical cord mesenchymal stromal cells.

Twenty-eight days after surgery (experimental day 0), proteinuria was quantified. Animals with similar levels of proteinuria, used as an indicator of the severity of CKD, were randomly allocated to five groups (*n* = 4 to 8 animals/groups) and received eight intravenous (i.v.) injections in the tail vein (on days 0, 3, 6, 9, 13, 16, 20, and 24) of 500 µl of saline, stromal cells or CM, as indicated in Fig. 1A. Group allocations were as follows: group 1, rats receiving i.v. saline; group 2, rats receiving i.v. 1.5 × 10^6^ uc-MSCs/injection; group 3, rats receiving i.v. 500 µl CM/injection obtained from uc-MSCs; group 4, rats receiving i.v. 1.5 × 10^6^ kPSCs/injection, and group 5, rats receiving 500 µl CM/injection obtained from kPSCs. No respiratory problems or animal mortality were observed following cell or CM injections. Untreated sham-operated rats served as controls (group 6) and were followed for the same period of time. Twenty-four-hour urine samples were collected using metabolic cages, and proteinuria was determined using the Coomassie method with a Cobas Mira autoanalyzer (Roche Diagnostics System, Basel, Switzerland). Serum and urinary creatinine were measured using an enzymatic method with a Cobas Mira autoanalyzer. Rats were weighed and sacrificed on experimental day 26 (Fig. 1A). Kidney tissues were harvested for histology and immunohistochemistry analysis.

### Human Stromal Cell Engraftment in the Kidney and Other Organs

The presence of human stromal cells in the kidney, lung, liver, and heart of RMR rats was determined as previously described^17^. Tissue sections were incubated for 1 hour with a 1% bovine serum albumin blocking solution, followed by incubation with Alexa Fluor 488 conjugated anti-human nuclear antigen (HNA) antibody (1:50; clone 235 -1, Merck Millipore) overnight at 4 °C. Cell nuclei were counterstained with 4’,6-diamidino-2-phenylindole (DAPI; Merck Millipore), and the tissue structures were marked using Rhodamine-labeled wheat germ agglutinin (WGA, 1:400; Vector Laboratories, Burlingame, CA, USA).

### Renal Morphology

Kidney samples from the remnant kidney were fixed in Duboscq-Brazil or formalin. Paraffin-embedded sections (3 µm) were stained with periodic acid-Schiff and assessed by light microscopy. The extent of GS was expressed as the percentage of glomeruli affected (%GS) in 50 to 60 glomeruli per rat. The extent of tubular damage was expressed with a cumulative score from 0 to 4 (0, no changes; 1, changes affecting ≤25% of the sample; 2, changes affecting 26% to 50% of the sample; 3, changes affecting 51% to 75% of the sample; 4, changes affecting 76% to 100% of the sample) which includes tubular atrophy and dilation. The number of tubular casts was counted per high-power field (HPF, ×20) in interstitial areas. Histologic sections were analyzed by the same pathologist who was blinded to the experimental conditions. Renal fibrosis was examined in paraffin-embedded sections stained with Sirius red. The percentage of total area positive for Sirius red staining was quantitated in 15 to 20 fields (HPF, ×20) per kidney using ImageJ 1.40 g software. Digitized images were dichotomized using a threshold for Sirius red staining, and the values were expressed as the percentage of staining per total field area.

### Immunohistochemistry

For immunofluorescence studies, sections (3 µm) from optimal cutting temperature (OCT) or periodate-lysine-paraformaldehyde (PLP)-fixed kidney specimens were processed as appropriate. Sections were incubated with the following primary antibodies: goat anti-nephrin (1:100, sc-19000; Santa Cruz Biotechnology, Heidelberg, Germany), rabbit anti-Wilm’s tumor 1 (WT1 1:30, sc-7385, Santa Cruz), mouse anti-endothelial cell antigen (RECA-1; 1:100, MCA970GA, Bio-Rad Laboratories, Hercules, CA, USA), rabbit anti-fibronectin (1:800, AB2040, Merck Millipore), rabbit anti-NG2 (1:100, AB 5320, Merck Millipore), and mouse anti-α-smooth muscle actin (SMA) (1:200, C6198, Sigma-Aldrich, St. Louis, MO, USA). Sections were then incubated with the appropriate secondary antibody (Jackson ImmunoResearch Laboratories, Cambridge, UK). Sections incubated with anti-WT1, NG2, and α-SMA antibodies were counterstained with fluorescein isothiocyanate (FITC)-labeled WGA (Vector Laboratories) and DAPI. Double and triple fluorescence labeling was analyzed with an inverted confocal laser-scanning microscope (LS 510 Meta; Zeiss) in 10 to 15 random images per section (*n* = 3 sections per kidney). Images were analyzed using ImageJ 1.40 g software. Digitized images were dichotomized using a threshold for staining, and the values were expressed as the percentage of staining per glomerulus or per total area of the acquired field, as appropriate. The number of WT-1 positive podocytes was quantified by acquiring at least 30 glomeruli/sections for each animal using confocal microscopy. The estimation of the average number of WT-1 positive cells was assessed using a stereological method of particle density proposed by Weibel^23^. As for apoptosis, renal sections were incubated with anti-cleaved caspase-3 antibody (1:50, 9664, Cell Signaling Technology, Leiden, The Netherlands) and the appropriate secondary antibody followed by FITC-labeled WGA lectin and DAPI. The expression of cleaved caspase-3 in glomerular and tubular compartments was quantified by the software ImageJ 1.40 g. Digitized images were binarized using a threshold, and the values were expressed as a percentage of area occupied by cleaved caspase-3 staining per glomerulus or per total area of the acquired field, as appropriate (*n* = 30 glomeruli/section or 15 randomly selected HPF, ×40).

To study inflammatory cell infiltrates, renal tissues were stained with mouse anti-CD4 (1:25, MCA55G, Bio-Rad Laboratories) or mouse anti-CD8 (1:100, 22071D, BD Biosciences, San Jose, CA, USA) antibodies, and the average number of CD4^+^ and CD8^+^ T cells was quantified in the renal interstitium. Infiltrating cells were counted in 15 randomly selected HPF (×40). To quantify macrophages/monocytes, formalin-fixed paraffin-embedded kidneys were subjected to antigen retrieval in a decloaking chamber with Rodent decloacker buffer (RD913 M, Biocare Medical, Pacheco, CA, USA) and then incubated with Peroxidazed 1 (PX968H, Biocare Medical). After blocking with rodent block R (RDR962G, Biocare Medical), sections were incubated with mouse antimacrophages/monocytes, clone ED1 (1:50, MAB1435, Merck Millipore) followed by mouse-on-rat horseradish peroxidase-Polymer (MRT621G, Biocare Medical). To visualize the staining, diaminobenzidine (BDB2004H, Biocare Medical) substrate solution was used. Slices were stained with Mayer’s hematoxylin (MHS80, Bioptica, Milan, Italy), mounted with Eukitt mounting medium (09-00250, Bioptica), and observed using light microscopy (ApoTome, Axio Imager Z2, Zeiss). Infiltrating cells were quantified in 15 randomly selected HPF (×40).

### Statistical Analysis

Results are expressed as mean ± SE. Data were analyzed using analysis of variance coupled with Dunnett’s or Tukey’s (proteinuria) post hoc analysis, as appropriate. The statistical significance level was defined as *P* < 0.05. Data analysis was performed using GraphPad Prism (GraphPad Prism Software, Inc., La Jolla, CA, USA).

## Results

### Effects of Nonrenal and Renal Stromal Cells or Their CM on Proteinuria and Serum Creatinine

The renoprotective effect of cell-based therapy with ucMSCs, kPSCs, or their respective CM (CM-ucMSCs and CM-kPSCs) was investigated and compared with saline treatment in rats with RMR and sham-operated controls with no RMR (Fig. 1A). By 28 days after RMR (day 0 of experimental treatment), all rats had developed proteinuria (Fig. 1B). Proteinuria increased progressively in RMR rats given saline over the experimental period (*P* < 0.05 day 14 vs day 0 and *P* < 0.01 day 26 vs day 14), and by day 26, urine protein excretion had increased fourfold compared with day 0 (*P* < 0.001). At this time point, proteinuria ratio levels in rats treated with ucMSCs and CM-ucMSCs were comparable to those in saline-treated rats (Fig. 1B). In contrast, treatment with kPSCs and CM-kPSCs significantly attenuated proteinuria. Similar results were observed when the urinary protein/creatinine ratio was analyzed (Table 1). Unlike the effect on proteinuria, serum creatinine remained significantly elevated in all RMR rats and was not affected by any of the treatments (Table 1). The body weight of animals with RMR was significantly lower than that of control rats, and no differences were observed between the RMR animals receiving cells or CM (Table 1).

**Table 1.** Urinary Protein/Creatinine Ratio, Serum Creatinine, and Body Weight in Control and RMR Rats Infused with Human ucMSCs, kPSCs, or the Corresponding CM.

	Urinary protein/creatinine	Serum creatinine (mg/dl)	Body weight (g)
Control	0.47 ± 0.01	0.23 ± 0.01	543.5 ± 18.16
RMR + saline	11.32 ± 1.89***	0.92 ± 0.17**	480.13 ± 8.73*
RMR + ucMSCs	12.20 ± 1.38***	0.93 ± 0.14*	476.25 ± 9.47***
RMR + CM-ucMSCs	7.74 ± 1.60*	0.87 ± 0.09*	433.50 ± 27.68*
RMR + kPSCs	5.03 ± 0.90°	0.96 ± 0.14**	453.40 ± 18.29**
RMR + CM-kPSCs	6.08 ± 1.21°	0.96 ± 0.09**	473.00 ± 12.04**

All the data refer to RMR rats at 26 days. Data are mean ± SE. *P < 0.05, **P < 0.01, and ***P < 0.001 versus control; °*P* < 0.05 versus saline.

CM: conditioned medium; kPSCs: kidney perivascular stromal cells; RMR: renal mass reduction; ucMSCs: umbilical cord mesenchymal stromal cells.

### Effects of Nonrenal and Renal Stromal Cells or Their CM on Glomerular Structure

The impact of stromal cell and CM treatments on glomerular fibrosis was assessed through histological grading of GS. Morphological analysis using light microscopy showed a significant increase in the percent of glomeruli with GS in RMR rats given saline compared with control rats (Fig. 1C, D). The degree of GS in RMR rats given saline was not different from that seen in rats treated with ucMSCs, CM-ucMSCs, or CM-kPSCs. In contrast, it was significantly lower after treatment with kPSCs (Fig. 1C, D). GS was further quantitated as the percentage of the glomerular area that stained positive for fibronectin, detected through immunofluorescence. The percentage of fibronectin staining was significantly greater and distributed differently in the glomeruli of RMR rats given saline compared with controls. A significant reduction in the deposition and distribution of fibronectin was observed in all treatment groups compared with RMR rats given saline, with the greatest effect seen in RMR rats treated with kPSCs (Fig. 1E, F).

**Figure 2.**
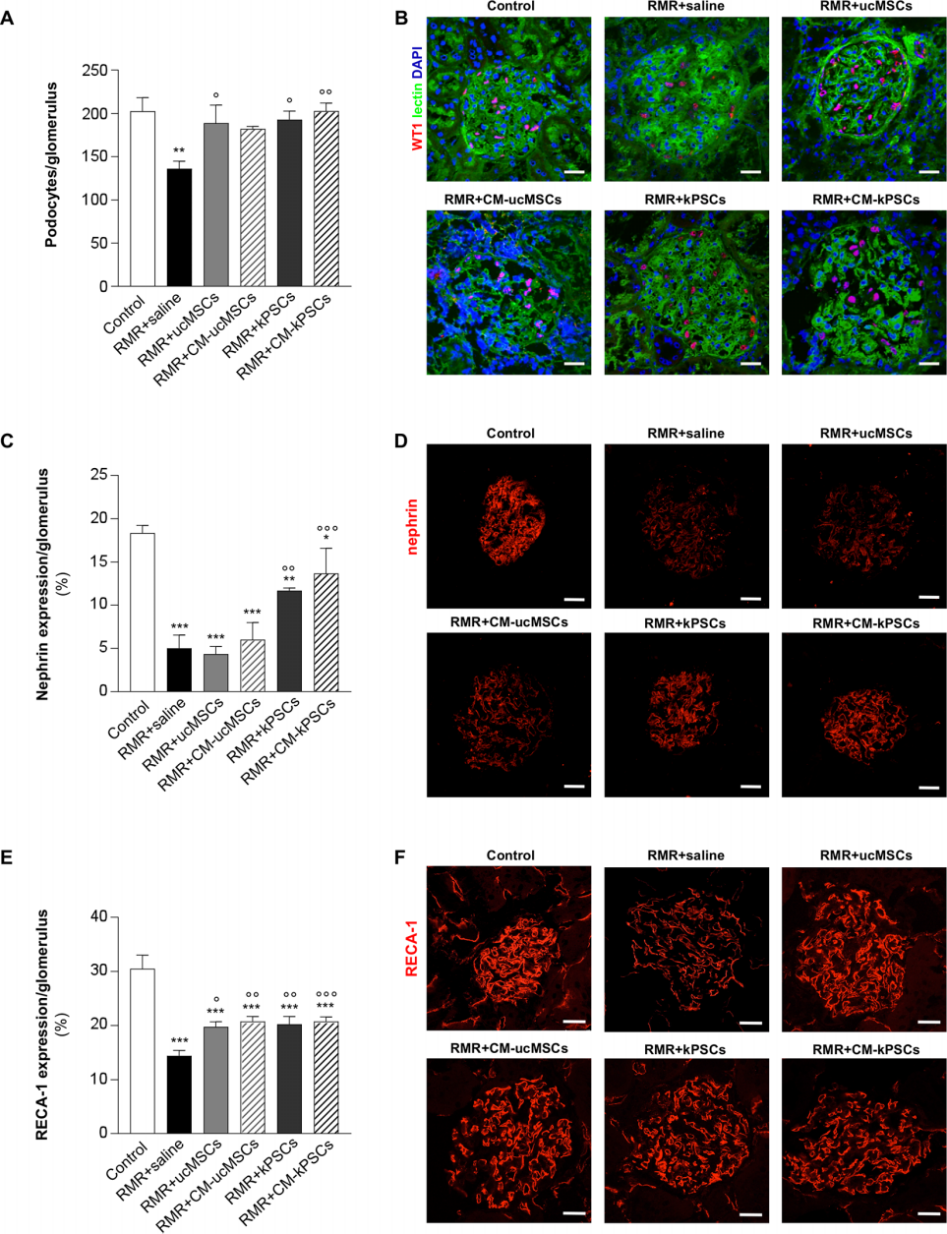
Effect of human ucMSCs, kPSCs, or their corresponding CM on glomerular podocyte and endothelial cell injury. (A) Quantification of the numbers of WT1-positive podocytes in control and RMR + saline, ucMSCs, kPSCs, or their corresponding CM on day 26. Data are mean ± SE. **P < 0.01 versus control; °*P* < 0.05 and °°*P* < 0.01 versus RMR + saline (*n* = 4 to 5 animals/group). (B) Representative micrographs of renal sections from control and RMR rats receiving saline, ucMSCs, kPSCs, or corresponding CM showing WT-1 positive podocytes (red). Renal structures were counterstained with WGA lectin (green) and nuclei with DAPI (blue). Scale bars 20 μm. (C) Quantification of the nephrin-positive area in control and RMR rats receiving saline, ucMSCs, kPSCs, or corresponding CM on day 26 (*n* = 4 animals/group). Data are mean ± SE. *P < 0.05, **P < 0.01, and ***P < 0.001 versus control; °°*P* < 0.01 and °°°*P* < 0.001 versus RMR + saline. (D) Representative micrographs of renal sections from control and RMR rats receiving saline, ucMSCs, kPSCs, or corresponding CM showing nephrin expression (red). Scale bars 25 μm. (E) Quantification of glomerular endothelial cells expressed as a percentage of glomerular area positive for RECA-1 staining in control and RMR rats receiving saline, ucMSCs, kPSCs, or CM at day 26 (*n* = 4 to 8 animals/group). Data are mean ± SE. ***P < 0.001 versus control; °*P* < 0.05, °°*P* < 0.01, and °°°*P* < 0.001 versus RMR + saline. (F) Representative images of glomerular endothelial cells stained with RECA-1 (red) in renal sections of control and RMR rats receiving saline, ucMSCs, kPSCs, or corresponding CM. Scale bars 25 μm. CM: conditioned medium; DAPI: 4′,6-diamidino-2-phenylindole; kPSCs: kidney perivascular stromal cells; RMR: renal mass reduction; ucMSCs: umbilical cord mesenchymal stromal cells; WGA: wheat germ agglutinin.

Podocyte injury and loss are involved in the progression of proteinuria in the RMR model. The quantification of WT-1 positive cells was used to determine podocyte loss (Fig. 2A, B). RMR induced a significant decrease in the number of WT-1 positive cells in RMR rats given saline compared with controls. Treatment with ucMSCs, kPSCs, and CM-kPSCs was associated with significantly greater numbers of WT-1 positive cells compared with saline treatment. WT-1 positive podocyte numbers in all cell-based therapy groups were no different from those in controls, indicating some protection from podocyte loss.

Nephrin expression was examined as a marker of podocyte integrity (Fig. 2C, D). Nephrin expression was lower in RMR rats receiving saline compared with controls. Expression was similarly reduced in rats treated with ucMSCs and CM-ucMSCs. In contrast, treatment with kPSCs and CM-kPSCs was associated with a significant increase in nephrin expression. Human kPSCs and CM-kPSCs therefore exerted a comparable protective effect on podocyte integrity.

Glomerular endothelial cell injury was assessed through RECA-1 expression (Fig. 2E, F). Morphometric analysis demonstrated a marked reduction in the RECA-1 positive area in RMR rats given saline compared with controls. All treatments were associated with a significant improvement in RECA-1 expression compared with saline treatment, although expression levels remained lower than those observed in controls. The analysis of cell apoptosis indicated that the percentage of cleaved caspase-3 positive area in glomeruli was higher in the renal tissue of RMR rats given saline compared with controls (percentage of cleaved caspase-3 positive area/glomerulus: control, 1.21 ± 0.08 vs RMR + saline, 4.65 ± 0.73; *P* < 0.001). Notably, all the treatments with cells or CM significantly decreased the expression of caspase-3 at the same extent (RMR + ucMSCs, 2.02 ± 0.08; RMR + CM-ucMSCs, 2.30 ± 0.16; RMR + kPSCs, 2.11± 0.22, and RMR + CM-KPSCs, 2.19± 0.12; *P* < 0.01 vs RMR + saline). These findings suggest that all stromal cells and CM treatments had a similar protective effect of the glomerular microvasculature after RMR.

### Effects of Stromal Cells or Their CM on Tubulointerstitial Structure

**Figure 3.**
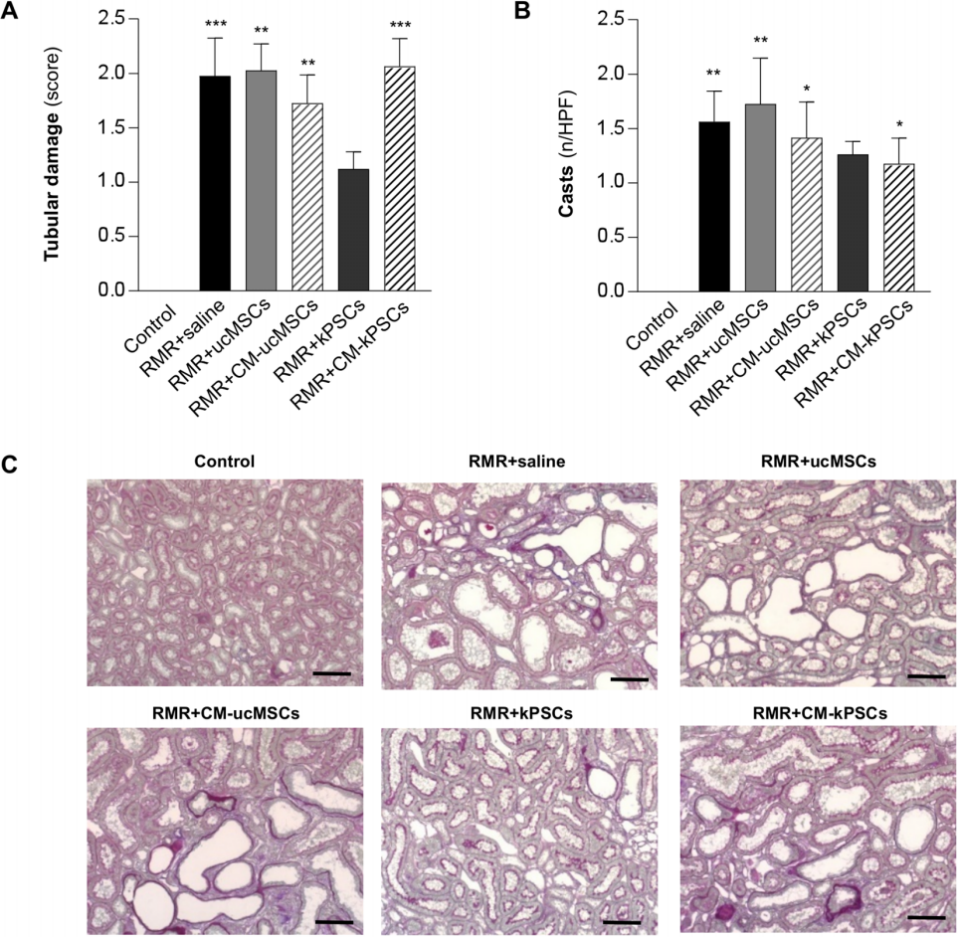
Effect of human ucMSCs, kPSCs, or their corresponding CM on renal tubular damage and casts. (A) Quantification of renal tubular damage expressed using a cumulative score from 0 to 4 (0, no changes; 1, changes affecting ≤25% of the sample; 2, changes affecting 26% to 50% of the sample; 3, changes affecting 51% to 75% of the sample; 4, changes affecting 76% to 100% of the sample), including tubular atrophy and dilation in control and RMR rats receiving saline, ucMSCs, kPSCs, or CM at day 26 (*n* = 4 to 8 animals/group). Data are mean ± SE. **P < 0.01 and ***P < 0.001 versus control. (B) The number of tubular casts counted per high-power field (×20) in interstitial areas (*n* = 4 to 8 animals/group). Data are mean ± SE. *P < 0.05 and **P < 0.01 versus control. (C) Representative images of PAS-stained sections of a kidney from control and RMR + saline, ucMSCs, kPSCs, or corresponding CM on day 26. Scale bars 100 μm. CM: conditioned medium; kPSCs: kidney perivascular stromal cells; RMR: renal mass reduction; ucMSCs: umbilical cord mesenchymal stromal cells.

The impact of stromal cell or CM treatments on tubulointerstitial injury and fibrosis was assessed through histological analysis (Fig. 3A–C). Kidneys from RMR rats given saline exhibited significant tubular injury, quantified using the tubular damage score and intratubular cast count, compared with the uninjured kidneys of control rats. Tubular injury was significantly higher in RMR rats given saline compared with control rats and was not reduced by all treatments, either cell therapy or CM, except for the kPSC group (*P* = 0.08 vs control).

**Figure 4.**
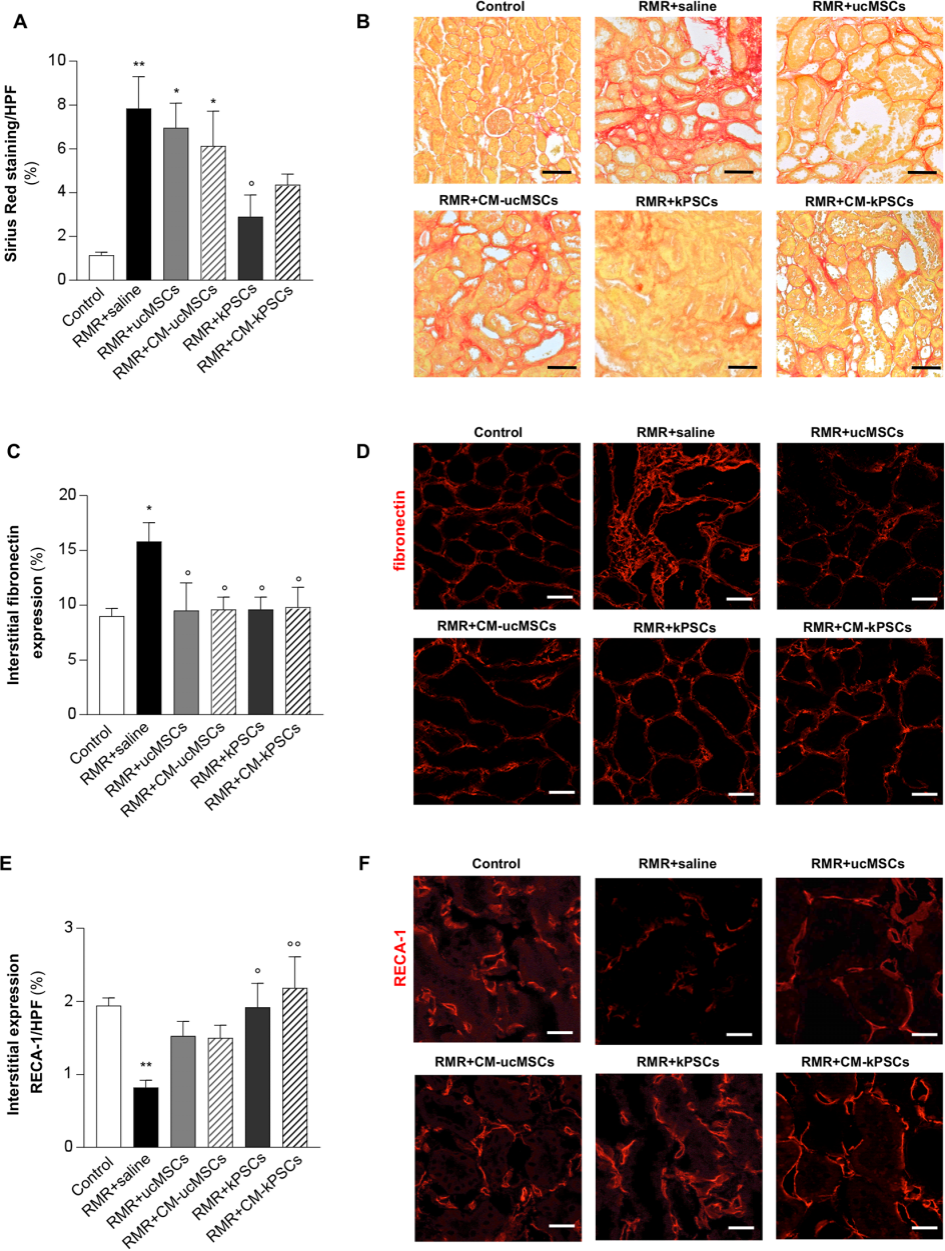
Effect of human ucMSCs, kPSCs, or their corresponding CM on tubulointerstitial fibrosis and peritubular capillary rarefaction. (A) Quantification of renal fibrosis evaluated as the percentage of positive area of Sirius red staining which reflects collagen deposition in renal tissue of control and RMR rats receiving saline, ucMSCs, kPSCs, or CM at day 26 (*n* = 4 to 6 animals/group). Data are mean ± SE. *P < 0.05 and **P < 0.01 versus control, °*P* < 0.05 versus RMR + saline. (B) Representative images of Sirius red-stained sections of kidneys from control and RMR rats receiving saline, ucMSCs, kPSCs, or CM. Scale bars 100 μm. (C) Percentage of interstitial fibronectin staining in the renal tissue of control and RMR-rats receiving saline, ucMSCs, kPSCs, or CM at day 26 (*n* = 4 to 5 animals/group). Data are mean ± SE. *P < 0.05 versus control; °*P* < 0.05 versus RMR + saline. (D) Representative micrographs of renal sections from control and RMR rats receiving saline, ucMSCs, kPSCs, or CM showing fibronectin expression (red). Scale bars 50 μm. (E) Quantification of peritubular microvascular endothelial cells expressed as the percentage of RECA-1 positive area/high-power field in control and RMR rats receiving saline, ucMSCs, kPSCs, or CM at day 26 (*n* = 4 to 6 animals/group). Data are mean ± SE. **P < 0.01 versus control; °*P* < 0.05 and °°*P* < 0.01 versus RMR + saline. (F) Representative micrographs of renal sections from control and RMR rats receiving saline, ucMSCs, kPSCs, or CM showing RECA-1 expression (red). Scale bars 20 μm. CM: conditioned medium; DAPI: 4′,6-diamidino-2-phenylindole; kPSCs: kidney perivascular stromal cells; RMR: renal mass reduction; ucMSCs: umbilical cord mesenchymal stromal cells; WGA: wheat germ agglutinin.

Tubulointerstitial fibrosis and renal collagen accumulation were quantitated as the percentage of Sirius red staining per HPF (Fig. 4A, B). The percentage of Sirius red staining was significantly higher in RMR rats given saline compared with controls. Treatment with ucMSCs and their CM did not affect tubulointerstitial fibrosis in RMR rats. A significant reduction in Sirius red staining was observed only after kPSC treatment compared with saline. However, a trend toward decreased staining was observed with CM-kPSCs, to the extent that they were not significantly different from control rats (*P* = 0.2), suggesting that these treatments had a positive effect on tubulointerstitial collagen accumulation in rat kidneys after RMR.

Renal fibrosis was also assessed through the quantitation of interstitial fibronectin expression (Fig. 4C, D). The percentage of interstitial area positive for fibronectin staining was significantly higher in RMR rats given saline compared with controls. In contrast, fibronectin expression was significantly lower in all treatment groups compared with the RMR saline group and was no different from the expression of controls (*P* = 0.99). All cell and CM treatments were therefore associated with the normalization of tubulointerstitial fibronectin expression suggesting a reduction in interstitial fibrosis after RMR.

Peritubular capillary density was assessed as a marker of vascular rarefaction through the quantification of RECA-1-positive areas in rat kidneys (Fig. 4E, F). RMR rats given saline exhibited significantly lower RECA-1 expression compared with controls. Treatment with kPSCs and CM-kPSCs significantly improved and normalized RECA-1 expression to control levels, whereas treatment with ucMSCs or CM-ucMSCs had no effect. Treatment with kPSCs and CM-kPSCs was therefore associated with a reduction in peritubular vascular rarefaction after RMR.

In the tubulointerstitial compartment, caspase-3 staining significantly increased in renal tissues of RMR rats given saline with respect to controls (percentage of cleaved caspase-3 positive area/HPF: control, 0.26 ± 0.01 vs RMR + saline, 5.08 ± 0.43; *P* < 0.001). RMR rats receiving cells or CM showed a significant decrease in the percentage of cleaved caspase-3 positive area (RMR + ucMSCs, 1.15 ± 0.53; RMR + CM-ucMSCs, 0.19 ± 0.14; RMR + kPSCs, 0.63 ± 0.25; and RMR + CM-KPSCs, 0.44 ± 0.15; *P* < 0.001 vs RMR + saline).

### Effects of Nonrenal and Renal Stromal Cells or Their CM on Pericyte Dysfunction

**Figure 5.**
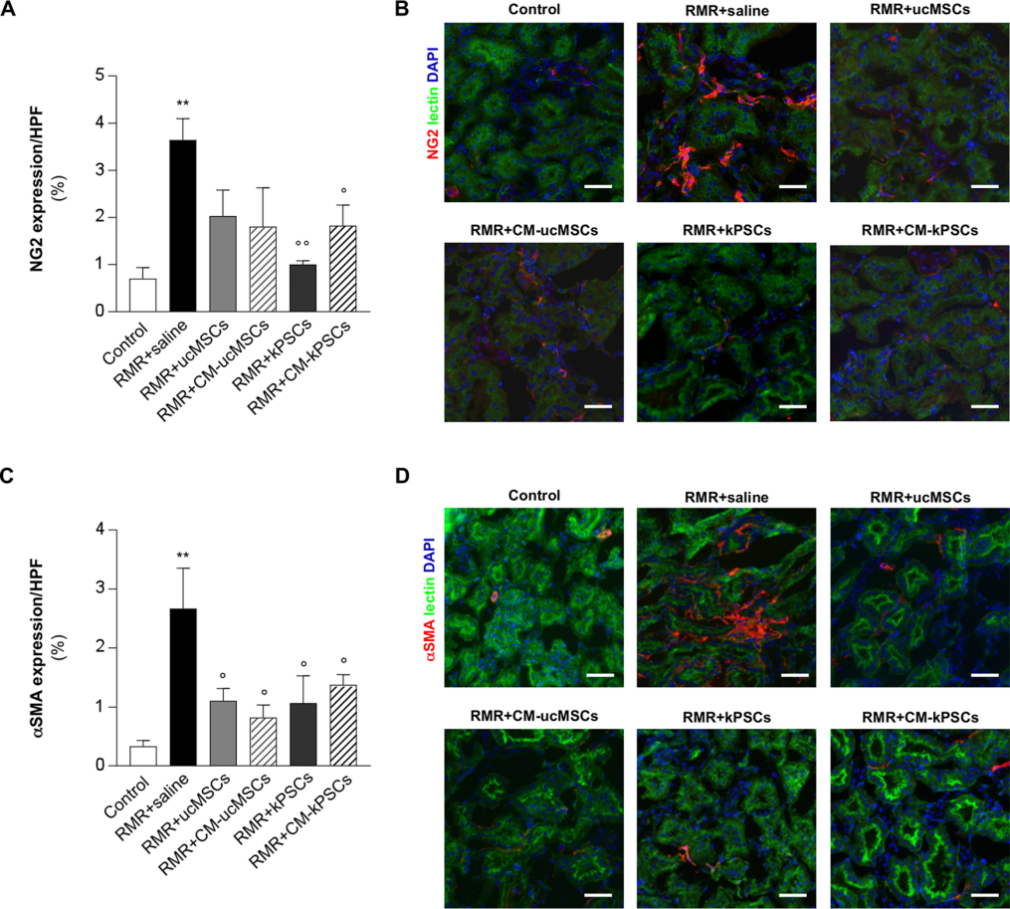
Effect of human ucMSCs, kPSCs, or the corresponding CM on pericyte dysfunction. (A) Quantification of pericyte activation evaluated as percentage of area positive for NG2 staining in renal tissue of control and RMR rats receiving saline, ucMSCs, kPSCs, or CM on day 26 (*n* = 4 to 5 animals/group). Data are mean ± SE. **P < 0.01 versus control; °*P* < 0.05 and °°*P* < 0.01 versus RMR + saline. (B) Representative images of interstitial cells stained for NG2 (red) and counterstained with WGA lectin (green) and nuclei with DAPI (blue) in renal sections of control and RMR rats receiving saline, ucMSCs, kPSCs, or CM. Scale bars 50 μm. (C) Quantification of the percentage of area positive for α-SMA staining in renal tissue of control and RMR rats receiving saline, ucMSCs, kPSCs, or CM on day 26 (*n* = 4 to 6 animals/group). Data are mean ± SE. **P < 0.01 versus control; °*P* < 0.05 versus RMR + saline. (D) Representative images of renal sections of control and RMR rats receiving saline, ucMSCs, kPSCs, or CM stained for α-SMA (red) and counterstained with WGA lectin (green) and nuclei with DAPI (blue). Scale bars 50 μm. α-SMA; alpha-smooth muscle actin; CM: conditioned medium; DAPI: 4′,6-diamidino-2-phenylindole; kPSCs: kidney perivascular stromal cells; RMR: renal mass reduction; ucMSCs: umbilical cord mesenchymal stromal cells; WGA: wheat germ agglutinin.

Pericytes are fibroblast-like cells involved in the development of interstitial fibrosis in renal parenchyma and play a crucial role in the onset and progression of renal fibrosis and peritubular microvascular rarefaction^24,25^. Renal expression of pericyte and myofibroblast markers, including chondroitin sulfate proteoglycan NG2 (Fig. 5A, B) and α-SMA (Fig. 5C, D), was analyzed through immunofluorescence. The expression of NG2-positive cells was significantly higher in RMR rats given saline compared with controls (Fig. 5A, B). Pericyte expansion was lower in all treatment groups but only reached statistical significance after treatment with kPSCs and CM-kPSCs. NG2-positive staining in all treatment groups was no different from controls (*P* > 0.2 for all groups), suggesting that pericyte expansion had been inhibited in all treatment groups. Similarly, a greater percentage of renal cells were positive for α-SMA, a marker of myofibroblast differentiation, in the tubulointerstitial compartment in RMR rats given saline than in controls (Fig. 5C, D). Treatment with ucMSCs, kPSCs, and their corresponding CM all resulted in a significant reduction in α-SMA expression, which approached control levels (*P* > 0.5 for all groups vs controls), suggesting the attenuation of pericyte expansion and a reduction in myofibroblast formation after RMR.

### Effect of Nonrenal and Renal Stromal Cells or Their CM on Inflammatory Cell Infiltration in the Kidney

The infiltration of CD4^+^ and CD8^+^ T cells was evaluated in renal tissues of RMR rats that received saline, cell treatment, or CM treatment. The analysis of kidney specimens from RMR rats that received saline showed an increased number of cells with CD4 or CD8 phenotype infiltrating the renal interstitium (Table 2). Treatments with ucMSCs, kPSCs, and their corresponding CM slightly decreased the average number of CD4^+^ and CD8^+^ T cells at the renal level, though not to a significant extent (Table 2). When we quantified the presence of infiltrating macrophages in the renal tissues of RMR rats, ED1 staining was significantly higher in rats that received saline compared with controls (Table 2). All treatments with cells and CM limited the number of ED1^+^ infiltrating cells slightly, but only rats given kPSCs had a significant reduction of interstitial macrophage infiltration compared with RMR rats given saline (Table 2). Notably, the ED1^+^ cells in the renal tissues of all animals that received cells or CM were not statistically different from controls, suggesting that these treatments had a positive effect on inflammatory cell accumulation in rat kidneys after RMR (Table 2).

**Table 2.** Renal Interstitial Accumulation of Inflammatory Cells in Control and RMR Rats Infused with Human ucMSCs, kPSCs, or the Corresponding CM.

	CD4^+^ T cells (cells/HPF)	CD8^+^ T cells (cells/HPF)	ED1^+^ cells (cells/HPF)
Control	12.92 ± 1.06	5.92 ± 0.33	0.00 ± 0.00
RMR + saline	65.95 ± 7.16***	31.05 ± 3.69***	11.20 ± 2.92**
RMR + ucMSCs	52.71 ± 6.22***	26.69 ± 2.41**	4.75 ± 1.93
RMR + CM-ucMSCs	54.40 ± 3.91***	24.43 ± 2.53**	5.33 ± 2.03
RMR + kPSCs	49.91 ± 3.34***	23.29 ± 2.20*	0.67 ± 0.33°
RMR + CM-kPSCs	52.20 ± 2.63***	26.61 ± 5.54**	5.33 ± 2.19

All the data refer to RMR rats at 26 days. Data are mean ± SE. *P < 0.05, **P < 0.01, and *** *****P***** < 0.001 versus control; °*P* < 0.05 versus saline.

CM: conditioned medium; HPF: high power field; kPSCs: kidney perivascular stromal cells; RMR: renal mass reduction; ucMSCs: umbilical cord mesenchymal stromal cells.

### Engraftment of Stromal Cells in Kidney Parenchyma and Other Organs

To understand the mechanisms underlying the potentially different impacts of ucMSCs and kPSCs, cell engraftment was analyzed in RMR kidneys, as kPSCs have been found to have specific renal tissue imprinting and were able to integrate into the neonatal kidney^15^. Engraftment was determined through the quantification of human cells positive for HNA. No HNA-positive cells, or very few, were observed in the kidneys of both ucMSC-treated and kPSC-treated rats, suggesting that the observed therapeutic effects were mediated via paracrine mechanisms. When we analyzed the engraftment of human ucMSCs or kPSCs in the lung, liver, and heart of RMR rats, no human cells positive for HNA were found.

## Discussion

We investigated the effects that different stromal cells, of nonrenal and renal origin, and their CM had on reducing the extent of kidney injury in a rat model of CKD. Therapy with kPSCs or CM-kPSCs induced the attenuation of proteinuria, likely mediated through the preservation of the glomerular structure and a marked protection against podocyte dysfunction/loss and peritubular microvascular rarefaction in rats with RMR. Treatment with kPSCs and the corresponding CM was also more effective than ucMSCs in reducing renal fibrosis and pericyte and myofibroblast expansion. Together, these findings suggest that kidney-derived stromal cell and CM therapies have the greatest potential to reduce renal injury in this CKD model.

Reducing proteinuria is an established therapeutic goal for the prevention of the progression of CKD^26–28^. The attenuation of proteinuria and urinary protein/creatinine ratio observed with kPSC therapy and its CM is consistent with the findings of other researchers, who used cells of various origins and at varying concentrations, although these were generally administered at earlier time points after the induction of RMR^29–31^. The efficacy of cell-based therapy in reducing protein excretion was also confirmed in a recent meta-analysis of animal studies using various CKD models, despite significant heterogeneity in study design, the origin of stem cells, cell number, timing and frequency of infusion, and route of delivery^16^. Thus, improvements in proteinuria after treatment with renal stromal cells or their CM appears to be relatively consistent.

Podocytes are pivotal cells that modulate proteinuria and stabilize the glomerular basement membrane and are therefore important target cells for the preservation of glomerular function. Treatment with both kPSCs and CM-kPSCs limited podocyte dysfunction/depletion and glomerular capillary rarefaction after RMR, as reflected by the relative preservation of WT-1, nephrin, and RECA-1 expression in glomeruli. Indeed, compared with saline or ucMSC treatment, proteinuria was halved or reduced by one-third after treatment with kPSCs and CM-kPSCs, respectively, consistent with the protective glomerular cell changes observed. Regarding glomerular fibrosis, fibronectin accumulation-an important indicator of the severity of RMR-was reduced markedly by all treatments, while glomerulosclerosis decreased only with the administration of kPSCs, consistent with the greater reduction in proteinuria.

Higher protection of kPSCs and their CM was also observed in the tubular compartment where these therapies have an important antifibrotic effect, possibly through the inhibition of the activation of pericytes/myofibroblasts which produce extracellular matrix proteins during chronic renal injury^24,32,33^. In the RMR model, a positive impact of treatment with a different type of stromal cell, bmMSCs, on renal fibrosis and/or fibronectin expression was also reported^30,31^.

The preservation of microvascular integrity is crucial to preventing the progression of tubulointerstitial fibrosis. Treatment with ucMSCs and CM-ucMSCs improved vascular rarefaction in the glomerulus but not in the tubulointerstitium. Treatment with kPSCs and CM-kPSCs was effective in both compartments and more effective than ucMSCs, suggesting that kPSCs and their secretome have higher specificity in the maintenance and stabilization of the peritubular microvasculature network in RMR animals, in line with previous *in vitro* data^15^. In this context, pericytes are involved in the regulation of microvascular integrity in the tubulointerstitial compartment^34^. These cells are physically in contact with the endothelium, embedded in the vascular basement membrane, and play a crucial role in kidney homeostasis regulating angiogenesis and vascular cell survival as well as blood flow^24,25^. The detachment of pericytes from the renal microvasculature and their activation/proliferation is detrimental for endothelial cells leading to renal fibrosis and tissue hypoxia^24,25,32^. These findings support our data which indicates that the detachment of these cells from the vascular wall and differentiation into myofibroblasts in response to RMR could lead to peritubular microvascular injury and interstitial fibrosis. Actually, the increased expression of NG2 and α-SMA in RMR rats given saline suggests the presence of an aberrant pericyte population in this model. Treatment with kPSCs and CM-kPSCs was associated with a reduction in NG2 expression, whereas α-SMA expression was reduced in all treatment groups. The above data could suggest that kPSCs and their CM exert a greater antifibrotic effect than ucMSCs, based on their ability to better counteract pericyte activation and their expansion than α-SMA positive cells, thus limiting the production and deposition of extracellular matrix proteins. Moreover, kPSCs also had grater anti-inflammatory effects compared with the other treatments, based on our finding that a significant reduction of macrophage accumulation was observed in the renal interstitium of RMR rats. Consistent with the reduction of inflammation and fibrosis in this model, here we also provide evidence that these cell-based or CM-based therapies had similar effects in reducing the expression of caspase-3, the key enzyme for the execution of the apoptotic program, both in the glomerular and tubulointerstitial compartment.

Importantly, we observed that cell therapy had beneficial effects despite very low engraftment in the damaged kidney, supporting the hypothesis that mediation occurs via paracrine activity. Consistent with this, the efficacy of human CM-kPSCs has been reported in a tubular epithelial wound scratch assay^15^. To the best of our knowledge, this is the first study to investigate the effects of CM-kPSCs in an *in vivo* model of CKD. We found that kPSCs and CM-kPSCs share similar renoprotective properties, such as reducing proteinuria and limiting podocyte injury and microvascular rarefaction. Some differential effects, for example, on collagen deposition in the tubulointerstitium and GS, however, suggest there are different mechanisms of interaction with resident cells. kPSCs, given their peculiar phenotype and their potent kidney epithelial wound-healing capacity, may interact more effectively with the tubular compartment than CM alone. However, earlier studies have shown that the administration of bioactive molecules secreted by stromal cells improved renal function and renal structural recovery in different CKD models^7,35^, and these effects may be mediated by secretomes and extracellular vesicles that contain mRNAs, miRNAs, or proteins^36,37^.

Differences in the secretome of the distinct stromal cell populations may account for their diversity and overall efficacy in the reparative response *in vivo* and, in our study, may explain why kPSCs are more effective than ucMSCs. Although a direct comparison with uc-MSCs was not studied, an initial analysis of the kPSC secretome has been performed to compare them with MSCs of bone marrow origin^15^ and showed there was a distinct, higher growth factor excretion of hepatocyte growth factor, which resulted in more potent wound-healing capacity^15^. Notably, stroma-derived factor 1, a factor that is important for kidney regeneration^38^, was produced in a concentration that was over 100-fold higher than that found in bmMSCs^39^. Studies have reported differences between bmMSCs and ucMSCs in secretome profile and have shown that ucMSCs have a higher capacity to release anti-inflammatory and proangiogenic molecules^40,41^. Nevertheless, here CM-kPSCs were even more therapeutically effective than CM-ucMSCs in the kidney, suggesting that the kPSC secretory profile is more suitable for kidney regeneration in this model of CKD. CM could therefore be a useful clinical tool because it is easier to scale up and has fewer potential safety and reproducibility concerns than cell therapy.

Our study has some limitations but also several strengths. Blood pressure was not measured, so we are unable to determine whether hemodynamic changes might have modified the observed effects on proteinuria and tissue injury. Others have described a blood pressure-lowering effect of bmMSC therapy in RMR rats. Future studies are required to investigate the hemodynamic impact of cell therapies and CM of various origins. Ultimately, however, independently on the mechanism through which stromal cells or CM therapies improve kidney injury, via local mechanisms alone or in conjunction with systemic changes, they promote the beneficial effects and therefore support the proof of principle in this study.

This study has several strengths. The 5/6 nephrectomy model of progressive kidney dysfunction is well characterized and used extensively. Functional and histological data were substantiated by immunohistochemical studies, and the stratification of findings by kidney compartment (glomerular, tubulointerstitium, and microcirculation) has not always been reported in earlier studies. Importantly, the differential effects of treatments on glomeruli and the tubulointerstitum were highlighted, clarifying the mechanisms underlying the superior effects of kPSC and CM therapy. In addition, to our knowledge, this is the first study that compares two different cell types and their CM and provides the first proof of principle that CM-kPSCs may be effective in the attenuation of injury in this model of CKD. Understanding the effectiveness of therapies with renal stromal cells and their CM, once the disease is already established, is clinically relevant, and important with a view to translation into real practice.

## Conclusion

With the present study, we provide evidence supporting the hypothesis that kPSCs and their corresponding CM have a nephroprotective effect in an established proteinuric model of CKD in rats. kPSCs and their CM significantly reduced proteinuria, possibly by limiting podocyte injury and loss. Similar protective effects were noted in the tubulointerstitium. kPSCs and, to a lesser extent, their CM, reduced renal microvascular rarefaction, apoptosis, fibrosis, and attenuated pericyte expansion, as well as inflammatory response. Treatment with these kidney-derived cell therapies may be superior to treatment with ucMSCs and their CM in RMR model of CKD. The effectiveness of CM therapy and lack of cell engraftment into kidney tissue suggest a predominantly paracrine mechanism of action. Further investigations, regarding the composition of CM-growth factors and microvesicles- from kPSCs and the identification of the precise mechanism for therapeutic effect, are needed to pave the way for future clinical applications.
